# PCR-RFLP Detection and Genogroup Identification of *Piscirickettsia salmonis* in Field Samples

**DOI:** 10.3390/pathogens9050358

**Published:** 2020-05-08

**Authors:** Pamela Aravena, Rodrigo Pulgar, Javiera Ortiz-Severín, Felipe Maza, Alexis Gaete, Sebastián Martínez, Ervin Serón, Mauricio González, Verónica Cambiazo

**Affiliations:** 1 Laboratorio de Bioinformática y Expresión Génica, Instituto de Nutrición y Tecnología de los Alimentos (INTA), Universidad de Chile, Santiago 7830490, Chile; paravena@inta.uchile.cl (P.A.); rpulgar@inta.uchile.cl (R.P.); javiera.ortiz@inta.uchile.cl (J.O.-S.); felipemaza.f@gmail.com (F.M.); alexis.gaete@inta.uchile.cl (A.G.); mgonzale@inta.uchile.cl (M.G.); 2 FONDAP Center for Genome Regulation, Santiago 8370415, Chile; 3 Laboratorio Especialidades Técnicas Marinas (ETECMA), Puerto Montt 5500001, Chile; sebastian.martinez@etecma.cl (S.M.); ervin.seron@etecma.cl (E.S.)

**Keywords:** *Piscirickettsia salmonis*, polymerase chain reaction-restriction fragment length polymorphism (PCR-RFLP), LF-89-like, EM-90-like, 16S rDNA sequencing

## Abstract

*Piscirickettsia salmons*, the causative agent of piscirickettsiosis, is genetically divided into two genomic groups, named after the reference strains as LF-89-like or EM-90-like. Phenotypic differences have been detected between the *P. salmonis* genogroups, including antibiotic susceptibilities, host specificities and pathogenicity. In this study, we aimed to develop a rapid, sensitive and cost-effective assay for the differentiation of the *P. salmonis* genogroups. Using an in silico analysis of the *P. salmonis* 16S rDNA digestion patterns, we have designed a genogroup-specific assay based on PCR-restriction fragment length polymorphism (RFLP). An experimental validation was carried out by comparing the restriction patterns of 13 *P. salmonis* strains and 57 field samples obtained from the tissues of dead or moribund fish. When the bacterial composition of a set of field samples, for which we detected mixtures of bacterial DNA, was analyzed by a high-throughput sequencing of the 16S rRNA gene amplicons, a diversity of taxa could be identified, including pathogenic and commensal bacteria. Despite the presence of mixtures of bacterial DNA, the characteristic digestion pattern of the *P. salmonis* genogroups could be detected in the field samples without the need of a microbiological culture and bacterial isolation.

## 1. Introduction

*P. salmonis* is the etiological agent of piscirickettsiosis, an aggressive disease that causes important economic losses in the salmon farming industry [1,2,3]. This bacterium produces a systemic infection characterized by the colonization of several organs including kidney, liver, spleen, intestine, brain, ovary and gills [4]. *P. salmonis* was initially isolated in 1989 from a moribund coho salmon (*Oncorhynchus kisutch*) during an epizootic event that took place in the south of Chile and was named as the *P. salmonis* LF-89 strain [5,6,7]. Since then, *P. salmonis* has caused serious losses in all farmed salmonid fish species including Atlantic salmon (*Salmo salar*), rainbow trout (*Oncorhynchus mykiss*), Chinook salmon (*Oncorhynchus tshawytscha*), pink salmon (*Oncorhynchus gorbuscha*) and masu salmon (*Oncorhynchus masou*) [6,8,9,10]. *P. salmonis* covers a wide geographic range and outbreaks of piscirickettsiosis have been reported among farmed salmonid in Canada, Norway and Ireland; however, mortalities have not been as high as those recorded in Chile [11]. For example, a 0.6–15% mortality rate has been reported in Canada, Norway and Scotland [9,12,13], whereas data from the Chilean National Fisheries and Aquaculture Service (Sernapesca) indicates that during 2018, up to 83.3% of the mortalities ascribed to infectious diseases in the main species of farmed salmonids were associated with piscirickettsiosis [3]. 

Field isolates of *P. salmonis* have shown variable levels of virulence that have been linked to the differences in their genetic backgrounds [14,15]. Genetic diversity among Chilean isolates of *P. salmonis* was first detected by sequencing the ribosomal genes of six *P. salmonis* isolates [16]. The results indicated that the Chilean isolate named EM-90 diverged genetically from the rest. Other studies based on genomic and phylogenetic analyses using 16S ribosomal DNA (rDNA), support the existence of two clusters among the *P. salmonis* strains that have been often named after the reference strains, LF-89 or EM-90 [17,18,19]. With recent genomic comparisons [20], it is now clear that *P. salmonis* strains can be split into two separate genogroups named here as LF-89-like and EM-90-like. The differences that have been reported between these genogroups include geographic distributions, antibiotic susceptibilities and host specificities [21]. Moreover, Rozas-Serri et al. [22] showed that the LF-89 reference strain and a member of the EM-90-like genogroup elicited different clinical signs in post-smolt Atlantic salmon, with the EM-90-like strain being the one that caused a higher cumulative mortality and a lower mean time to mortality when compared with the LF-89 strain, suggesting pathogenic differences between the *P. salmonis* genogroups. 

Historically, the identification of *P. salmonis* in infected fish tissues has depended on phenotypic and molecular methods such as the culture of *P. salmonis* on a nutritive media followed by the Gram and Giemsa stain technique [23,24,25], conventional PCR [26,27], real-time PCR [28,29] and indirect fluorescence antibody testing [30]. Even though all of these methods are currently in use to detect the presence of *P. salmonis* in infected fish tissues, none of them can guarantee the unique presence of the bacterium in the tissue samples [18]. Furthermore, these methods were not designed to discriminate between the LF-89-like or EM-90-like genogroups, a distinction that has become increasingly necessary due to the phenotypic differences detected between the genogroups. These differences might be translated into different pathogenic profiles and also into immunogenic dissimilarities between the genogroups, as it has been reported for other pathogens of clinical relevance [31,32]. Thus, current diagnostic approaches need to be updated to improve the effectiveness of piscirickettsiosis control protocols, which heavily rely on antibiotic treatments and vaccinations. For the identification of the *P. salmonis* genogroups, an alternative method is the DNA sequencing of the rRNA gene portions, which is usually performed by PCR amplification followed by Sanger sequencing. While this approach is highly reliable and discriminatory, it is time-consuming and costly, and therefore not suitable for most diagnostic laboratories, especially when several hundred clinical isolates are to be identified. For reference laboratories or large epidemiologic studies, a more rapid and inexpensive approach may be required to discriminate between the *P. salmonis* genogroups. 

In this study, we developed a new PCR-RFLP (polymerase chain reaction-restriction fragment length polymorphism) assay based on universal 16S rRNA primers and the restriction enzyme *Xap*I for the amplification and digestion steps, respectively. This PCR-RFLP is a rapid, sensitive and cost-effective method to differentiate between the LF-89-like and EM-90-like genogroups. This assay was initially validated by comparing the restriction patterns of 13 *P. salmonis* strains and further validated by analyzing 57 unknown field samples. Our results showed the PCR-RFLP assay to be a useful tool to identify the *P. salmonis* genogroups in the field tissue samples without the need of microbiological cultures and bacterial isolation.

## 2. Results

### 2.1. Design of a PCR-RFLP Assay to Discriminate between P. salmonis Genogroups

**Figure 1:**
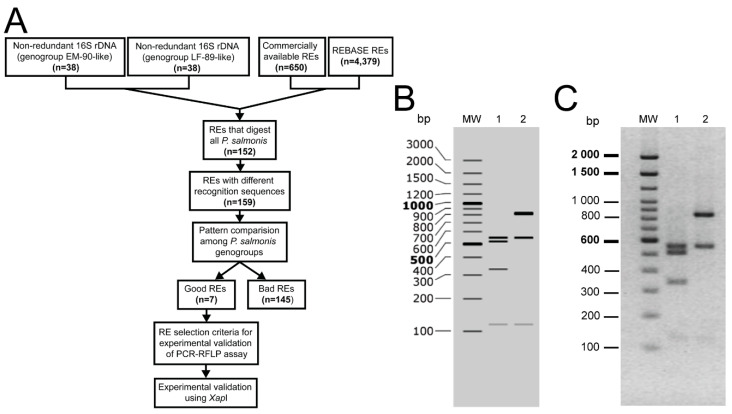
Polymerase chain reaction-restriction fragment length polymorphism (PCR-RFLP) assay discriminates between the *P. salmonis* LF-89-like and EM-90-like genogroups. (**A**) Bioinformatics pipeline for the selection of restriction enzymes that differentiate among the genogroups of *P. salmonis* in a PCR-RFLP assay. REs: Restriction enzymes. Good REs: REs that produce differentiable patterns between the genogroups. Bad REs: REs that do not produce differentiable patterns between the genogroups of *P. salmonis*. (**B**) Predicted digestion patterns of the 16S rRNA gene using SnapGene (version 4.2.6) for the reference strains LF-89 (lane 1) and EM-90 (lane 2). Molecular weight (MW): O’GeneRuler 100 bp Plus DNA Ladder (Thermo Fisher). (**C**) 2% gel electrophoresis showing the PCR-RFLP patterns of the LF-89 (lane 1) and EM-90 (lane 2) 16S rDNA fragments digested with the *Xap*I restriction enzyme. MW: 100 bp DNA Ladder (Invitrogen).

From the complete set of the 16S rDNA sequences of *P. salmonis* available at public databases (March 2020), we selected 76 complete non-redundant 16S rDNA sequences to develop a PCR-RFLP method for the specific identification of the *P. salmonis* genogroups. Thirty-eight of the sequences were representatives of the EM-90-like genotype and 38 were representatives of the LF-89-like genotype. To select an appropriate restriction enzyme for the PCR-RFLP assay, we follow the pipeline shown in Figure 1A. Starting with the 650 commercially available enzymes present at REBASE, we ended up with only seven enzymes, *Xap*I, *SfaN*I, *Hin4*II, *MluC*I, *Aci*I, *Fai*I and *Set*I, which produced predicted banding patterns that enabled us to distinguish between the genotypes (Figure 1A, Supplementary Table S1). Then, the *in silico* digestion patterns of the seven selected restriction enzymes were analyzed to decide on the better-suited enzyme for the PCR-RFLP typing assay. The enzyme that displayed the lowest number of bands and an easily discernible pattern that enabled to discriminate between the genogroups was *Xap*I (Figure 1B). Besides, this enzyme has an isoschizomer (*Apo*I), which could be relevant for its commercial availability and mass use. This enzyme generates three informative and easily recognizable bands in the 16S rRNA gene fragment of the EM-90-like strains (840, 548 and 119 bp) and four in the 16S rDNA gene fragment of the LF-89-like strains (548, 509, 331 and 119 bp). Upon gel electrophoresis, the RFLPs obtained for the reference strains LF-89 and EM-90 showed perfect matches with those predicted by the in silico digestion with the *Xap*I enzyme (Figure 1C). 

### 2.2. A T928G Transversion in the 16S rDNA of P. salmonis Enabled Differentiation between Genogroups

**Figure 2:**
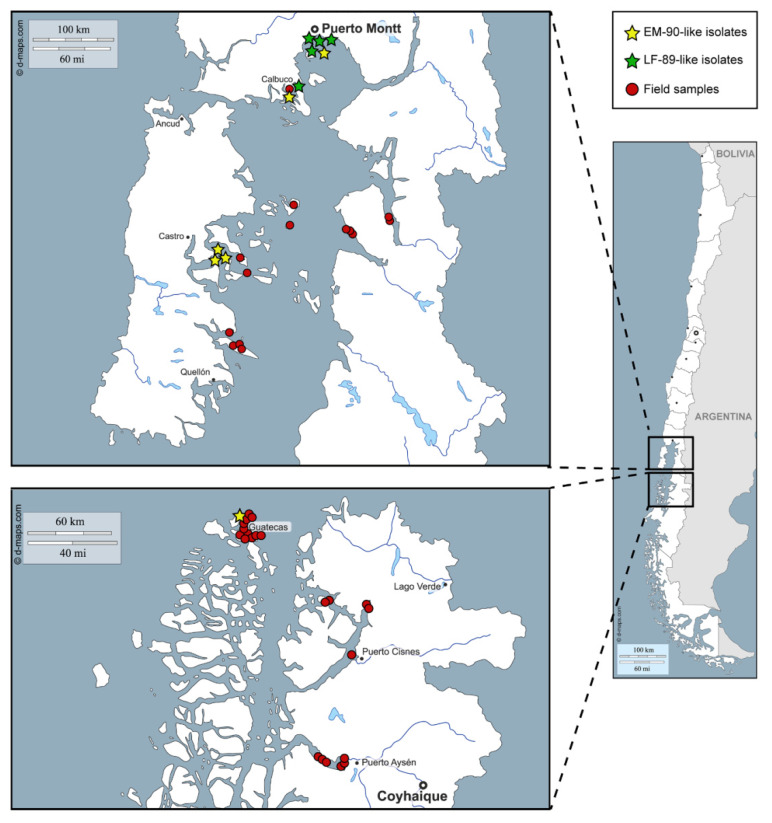
Geographical distribution of *P. salmonis* strains and field samples in southern Chile.

**Figure 3:**
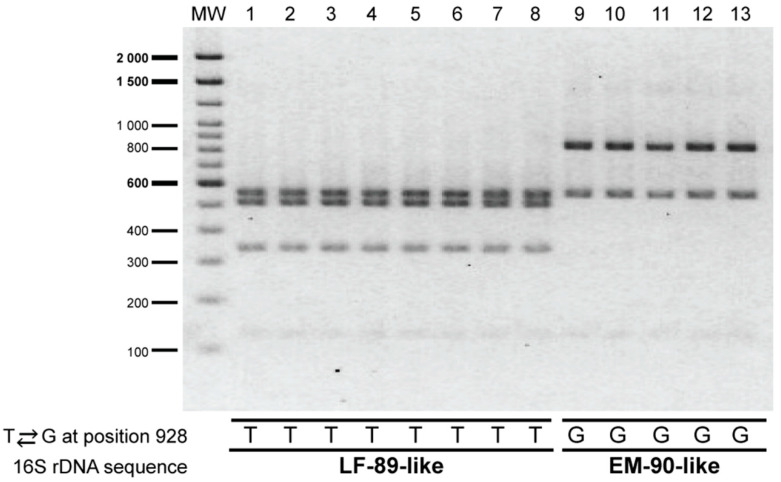
Evaluation of the PCR-RFLP assay on the selected *P. salmonis* strains. PsCGR01 (lane 1), CGR02 (lane 2), Ps8942B (lane 3), Ps11091B (lane 4), Ps16557B (lane 5), NVI5692 (lane 6), NVI5892 (lane 7), NVI5896 (lane 8), Ps8079A (lane 9), Ps2192A (lane 10), Ps12201A (lane 11), Ps18627A (lane 12) and Ps19647A (lane 13). Molecular weight (MW): 100 bp DNA Ladder (Invitrogen). The 16S rDNA sequence revealed a T ⇄ G transversion at position 928 that enabled to discriminate between the genogroups.

The PCR-RFLP assay was evaluated on 13 strains of *P. salmonis* that were isolated between years 2010 and 2018 from *P. salmonis* outbreaks that occurred in southern Chile (Figure 2, stars); most strains were obtained from *Salmo salar*, although two of them, Ps1191B and PSCGR01, were obtained from *Oncorhynchus kisutch* and *Oncorhynchus mykiss*, respectively. Initially, to determine which genogroup each strain belonged to, the 16S rRNA gene of the strains was sequenced using the universal primers 27F and 1492R, which span nearly the full-length of the 16S rRNA gene (1507 bp, Supplementary Figure S2A and Table S2). A comparison of the 16S rRNA gene sequences from the 13 strains and the reference strains LF-89 and EM-90 revealed a high level of sequence similarity (99%); however, 12 nucleotide differences were detected within the 16S rRNA gene sequences of all the strains examined (Supplementary Figure S1). All of the nucleotide variations were due to point mutations that covered 0.88% (12/1354 bp) of the alignment length. Among the 12 nucleotide differences, one was not genogroup-specific: a transversion located at position 128 (T ⇄ A). The other 11 genogroup-specific nucleotides found in the 16S rRNA gene of *P. salmonis* are located at positions 301, 928, 930, 931, 932, 933, 940, 941, 942, 945 and 1061. The T ⇄ G transversion located at position 928 was key to differentiate between the genogroups: strains with a T in that position belong to the LF-89-like genogroup, and strains with a G in that position to the EM-90-like genogroup. The presence of thymine in that location allows for a third cleavage with *Xap*I, generating a different RFLP pattern for the LF 89-like strains (Figure 3).

As expected from the 16S rRNA gene sequence analysis, the PCR-RFLP assay with *Xap*I applied to the 13 *P. salmonis* strains produced two different restriction patterns. Thus, five out of 13 strains yielded the restriction pattern specific for the EM-90-like genogroup and eight strains yielded the restriction pattern specific for the LF-89-like genogroup (Figure 3). Thus, the experimental approach using *Xap*I confirmed the *in silico* digestion patterns of *P. salmonis* 16S rRNA gene. 

### 2.3. Detection of P. salmonis Genogroups in Field Samples

**Figure 4:**
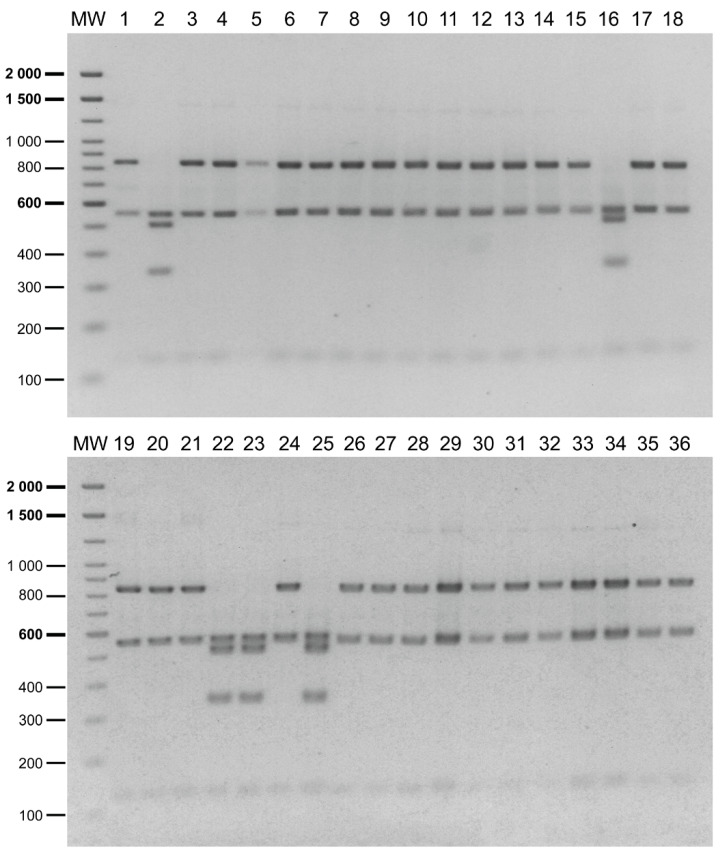
PCR-RFLP analysis of the field samples. Lanes 1 to 36 correspond to the restriction patterns of the 16S rRNA gene fragment amplified from the total DNA (fish plus pathogen DNA) extracted from moribund fish tissue and digested with *Xap*I. Restriction patterns characteristic of *P. salmonis* LF-89-like in lanes 2, 16, 22, 23 and 25; restriction patterns characteristic of *P. salmonis* EM-90-like in the remaining lanes. Molecular weight (MW): 100 bp DNA Ladder (Invitrogen).

The reliability of the PCR-RFLP to discriminate between the *P. salmonis* genogroups was further evaluated in the field samples (N = 61) obtained from the tissues of dead or moribund fish with clinical sinology consistent with piscirickettsiosis. The tissues from the infected fish were collected in seawater farms located in three southern regions of Chile (Figure 2, red circles, and Supplementary Table S3), mainly during the year 2019. After the DNA extraction from the tissue samples, a 16S rDNA fragment was successfully amplified in 57 field samples (a representative gel image in Supplementary Figure S2B) and the presence and purity of *P. salmonis* in the samples were tested by digestion with the *PmaC*I enzyme [18]. The results indicated that in 36 out of 57 (63%) field samples, the *PmaC*I digestion generated the expected electrophoretic pattern of four bands (732, 395, 281 and 97 bp), which was previously reported as specific for the *P. salmonis* 16S rDNA [18]. For these 36 samples, the digestion of the 16S rRNA gene fragment with the *Xap*I enzyme resulted in 31 patterns characteristic of the EM-90-like strains and five patterns characteristic of the LF-89-like strains (Figure 4). In the remaining 21 samples, additional bands were observed after the *PmaC*I (Supplementary Figure S3A) and *Xap*I digestions (Supplementary Figure S3B), indicating that, in addition to *P. salmonis*, other bacteria were present in those samples. 

### 2.4. Phylogenetic Analysis of the Field Samples 16S rDNA Sequences

**Figure 5:**
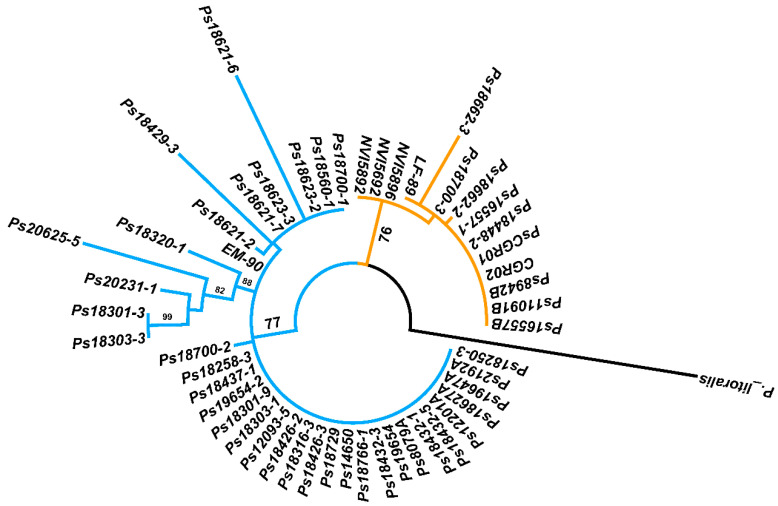
Phylogenetic tree constructed through a maximum likelihood analysis based on the 16S rDNA sequences. Bootstrap values ≥70% are indicated.

After the genogroup differentiation with the PCR-RFLP analysis, the 16S rRNA gene of the 36 field samples was amplified and sequenced and the resulting sequences were submitted to the GenBank under the accession numbers provided in Supplementary Table S3. A phylogenetic analysis based on the Tamura–Nei model [33] of maximum likelihood using near full-length 16S rRNA gene sequences (1345 bp) of the 13 *P. salmonis* strains plus the reference strains and the 36 field samples, separated the two *P. salmonis* genogroups (Figure 5), LF-89-like and EM-90-like, confirming the results of the PCR-RFLP analysis. The levels of identity of the 16S rDNA sequences within each genogroup were high. The LF-89-like cluster groups 14 isolates and field samples, within this cluster two sub-clusters were distinguished: one grouping of five Chilean isolates and four field samples together with the reference strain LF-89 (exhibiting reciprocal identity of the 16S rDNA sequences between 99.3% and 100%) and another clustering the Canadian and Irish isolates NVI5692, NVI5892 and NVI5896 (identity level 99.9%). The EM-90-like cluster also resolves into two main sub-clusters, one that groups 23 isolates and field samples exhibiting a high reciprocal identity (between 99.3% and 100%) of their 16S rDNA sequences, while the second sub-cluster gathers the reference strain EM-90 and 13 field samples that diverged as a separate branch in this sub-cluster, showing a reciprocal identity of the 16S rDNA sequences between 97.5% and 100 %. 

### 2.5. Bacterial Diversity in Field Samples Assessed by High-Throughput 16S Sequencing

**Figure 6:**
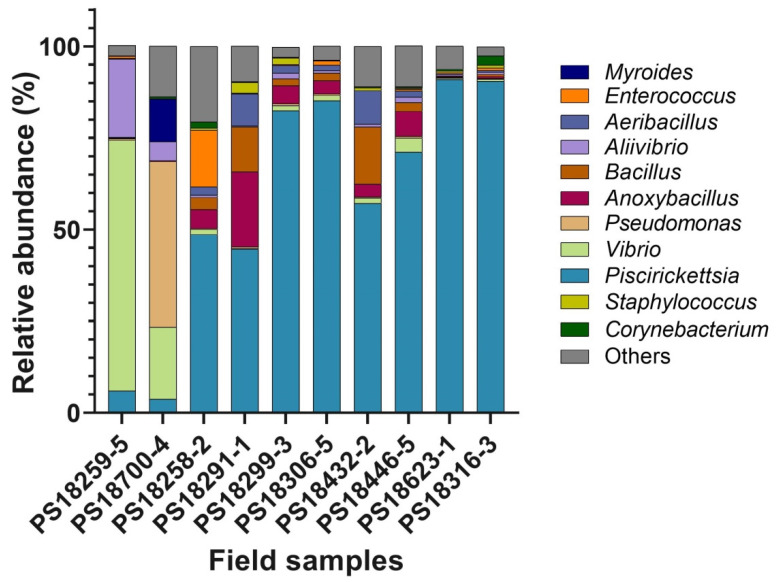
Bacterial taxa in the field samples. Stacked bar chart showing the relative abundance of the bacterial genera in the different field samples based on high-throughput sequencing of the V1-V3 hypervariable regions of bacterial 16S rRNA gene. The graph represents genera with a ≥ 1% relative abundance.

The field samples resolved as containing mixtures of bacterial DNA showed the characteristic *PmaC*I and *Xap*I digestion patterns of the *P. salmonis* 16S rRNA gene, but at least one additional band was also detected (Supplementary Figure S3A,B). We selected a representative subset of nine field samples to further analyze their bacterial contents by high-throughput sequencing of the V1–V3 hypervariable regions of the 16S rRNA gene and compared them with the bacteria present in a field sample in which only the characteristic *P. salmonis* restriction pattern was detected after the *PmaC*I digestion. An analysis of the raw sequence data yielded 269,868 reads after quality trimming. Taking together the 10 samples, we identified 513 operational taxonomic units (OTUs) (Supplementary Table S4) using a 95% identity threshold against the SILVA database. These OTUs were affiliated with 141 genera (Supplementary Table S4). Eleven genera exhibited ≥1.0% of relative abundance in the samples (Figure 6), with *Piscirickettsia* being the most prevalent genus (58% of the total relative abundance). In sample PS18259-5 (Figure 6), the 16S rDNA sequencing identified potential fish bacterial pathogens represented by the *Vibrio* and *Aliibrivio* genera, which accounted for 68.4% and 21.4%, respectively, of the relative abundance in that sample, while OTUs belonging to the *Piscirickettsia* genus accounted only for 6%. Similarly, in sample PS18700-4 (Figure 6), the most abundant genera were *Pseudomonas* (45.3%) and *Vibrio* (19.5%), whereas the *Piscirickettsia* genus was less abundant (3.8%). In the rest of the samples, the most abundant OTUs belonged to the *Piscirickettsia* genus, although variable percentages of OTUs belonging to other genera were also detected. The *Aeribacillus*, *Anoxybacillus*, *Pseudomonas, Vibrio, Corynebacterium* and *Aliibrivio* genera were associated with all the field samples and accounted for 25.8% of the relative abundance in the 10 samples. 

## 3. Discussion

In this study, we successfully developed a simple genotyping method to identify the genogroups of *P. salmonis*. Our PCR-RFLP assay is a rapid and cost-effective method, which allows to clearly differentiate the genogroups in the *P. salmonis* strains and also in the field samples. *P. salmonis* genotyping could be challenging when relying on isolates since the slow growth rate of *P. salmonis* in artificial media requires that isolates be cultured for several days to visualize the colonies. Moreover, culture media used to grow *P. salmonis* are highly nutritive and non-selective, thus they can easily be contaminated with microorganisms (bacteria and fungi) from the environment or those already present in the fish tissues. 

Previous genotyping methods aiming to differentiate between the *P. salmonis* genogroups relied on the Multilocus sequence typing (MLST) system based on the genetic variation present in 10 *P. salmonis* housekeeping loci [19,34]. This typing system allowed the separation of 18 Chilean *P. salmonis* isolates into two groups that were genetically distinct from the Canadian isolates, and confirmed the existence of at least two different *P. salmonis* genogroups. In that work, bacteria were isolated from sub-acute or chronic outbreaks of piscirickettsiosis that took place between the years 2011 and 2012 from two geographical areas, Puerto Montt and the east coast of Chiloe Island [19]. Recently, Isla et al. [34] reported an MLST for 42 *P. salmonis* isolates recovered from *Salmo salar*, *Oncorhynchus kisutch* and *Oncorhynchus mykiss* from two Chilean regions between the years 2008 and 2015. Their results supported the existence of two *P. salmonis* genetic groups, but also described possible divergent clades. While MLST is considered to be a highly reliable and discriminatory typing procedure, it is technically demanding, time-consuming and costly [35]. Hence, MLST may not be suitable for most microbiology laboratories, especially when several hundred field isolates are to be identified. For reference laboratories or large epidemiologic studies, a more rapid and inexpensive approach may be required to discriminate between the genogroups. To study the bacterial diversity, host-associated strains and virulence related to the *P. salmonis* genogroups, the present PCR-RFLP assay based on universal primers and the restriction enzyme *Xap*I provides a method that can be applied to a large number of samples during annual outbreaks without the need of microbiological cultures.

Even though all field samples were initially confirmed as positive to *P. salmonis* by the method described by Corbeil et al. [28], we further tested the purity of *P. salmonis* in the samples using a previously reported PCR-RFLP assay [18]. The results showed that 21 out of 57 field samples contained mixtures of bacterial DNA, suggesting that coinfection events with other fish pathogens or contamination with commensal/environmental microbiota occurred during the tissue dissection. Nevertheless, *P. salmonis* was present in those samples since the *PmaC*I digestion still revealed the characteristic pattern of this bacterium and the *Xap*I digestion generated the characteristic EM-90-like pattern (Supplementary Figure S3A,B, asterisks). The PCR-RFLP assay presented here appears to be able to identify the *P. salmonis* genogroups in the field samples in which this bacterium could not be the only species present. However, in those samples, the detection of the *P. salmonis* genogroups would require a comparison with the digested DNA of the reference strains. In addition, these results indicate that a direct 16S rRNA gene amplification and Sanger sequencing of the field samples would yield ambiguous results due to the mixed DNA chromatograms. Therefore, besides being more time consuming and costly, direct 16S rRNA gene sequencing does not seem to be a good alternative to distinguish between the *P. salmonis* genogroups in field samples. 

To find out the nature and, more importantly, the relative abundance of the different DNA species in the field samples, we further analyzed a representative sub-set of them by a culture-independent method. A high-throughput sequencing of the 16S rDNA amplicons was carried out to investigate the bacterial taxonomic diversity in the infected fish tissues from the field samples. We selected field samples that showed discernible *P. salmonis* restriction patterns but also additional bands after the *PmaC*I and *Xap*I enzyme digestions, as well as one control sample in which only the *P. salmonis* restriction pattern was detected after the *PmaC*I digestion. For the genera that exhibited ≥1.0% of relative abundance, we analyzed their taxa composition in the sequenced samples to discern their origin, whether commensal or pathogenic. In two of the nine samples analyzed (PS18259-5 and PS18700-4 in Figure 6), pathogenic bacterial genera such as *Vibrio*, *Aliivibrio*, *Pseudomonas*, and *Myroides* were predominant, suggesting potential co-infection events by either simultaneous or secondary infections with different pathogens. Reports on natural bacterial co-infection of fish are scarce, although *Aliivibrio wodanis* and *Moritella viscosa* have been concurrently isolated from the external surfaces and internal organs of fish with winter-ulcer disease [36]. On the other hand, bacteria belonging to 10 different genera, have been isolated from the kidneys of infected Chinook salmon, *Oncorhynchus tshawytscha* [37]. The authors reported a non-random association between the infections by *R. salmoninarum* and the motile members of the genus *Aeromonas*. As *R. salmoninarum* infection induces host immunosuppression, it has been suggested that the *Aeromonas* spp. act as an opportunistic pathogen that interact synergistically with *R. salmoninarum*. Here, the presence of different potential infectious agents was detected by a high-throughput sequencing of the tissue samples of infected fish; however, the possible interactions between these two pathogens, either synergistic or antagonistic, need to be investigated. *Vibrio* and *Aliivibrio* have been identified as members of the Atlantic salmon commensal microbiota [38]; however, a disturbance by a primary pathogen such as *P. salmonis* could have triggered an imbalance of the commensal microbial community, allowing the invasion of potential secondary bacterial pathogens [39]. 

In the rest of the field samples, the most abundant genus was *Piscirickettsia*, while other less abundant genera were also present even in sample PS18316-3 that was included as a control (Figure 6). Beside *Vibrio* and *Aliivibrio*, several genera previously identified as members of the commensal microbiota were also detected in those samples. For example, the genus *Aeribacillus*, which was identified as part of the skin microbiota of several fish species [40], and the genus *Anoxybacillus*, reported as part of the mucosal microbiota of brook trout [41]. The *Anoxybacillus* genus comprises cellulose-decomposing bacteria, which have been reported as part of the fecal bacterial communities of farmed Atlantic salmon [38]. Genera such as *Pseudomonas*, *Corynebacterium* and *Enterococcus* have also been detected as part of the gut microbiota [42,43,44] and in the fecal microbiome of Atlantic salmon [38]. 

Although care was taken to avoid contamination during the tissue sampling, we cannot discard that contamination could explain at least part of the bacterial diversity observed in these samples. Nonetheless, previous works have identified a wide range of bacteria in the kidney and liver of fish, even in allegedly healthy fish [36,45,46,47,48]. The presence of microorganisms in the internal organs of fish is not fully understood, although it has been interpreted as indicative of a breakdown of the immunological defense mechanisms [48], possibly due to the stress factors faced in aquaculture facilities [49]. In our case, whether a bacterial presence in the internal organs preceded the *P. salmonis* invasion or not needs to be determined.

Regarding the correlation between the *P. salmonis* genogroup and host species, Saavedra et al. [21] reported that the EM-90-like strains were exclusively isolated from *S. salar*, whereas the LF-89-like strains did not show host preference. Similarly, Isla et al. [34] noted that a higher proportion of the EM-90-like strains were isolated from *S. salar* than from *O. kisutch*. In agreement with those previous data, in 31 out of 36 field samples analyzed in this study, the genogroup of *P. salmonis* was resolved as EM-90-like, and 34 of these field samples were obtained from moribund specimens of *S. salar* and only two from *O. kisutch*. These results suggest that the EM-90-like *P. salmonis* are more often diagnosed in field cases of piscirickettsiosis among Chilean salmon farms, at least for those located at the geographical areas sampled in this work. Since most of the field samples were collected from diseased fish at the beginning of the year 2019 (29/36), it is tempting to speculate that the EM-90-like *P. salmonis* has become more prevalent than the LF-89-like genogroup. However, further analyses need to be performed, such as genotyping samples along an entire year, sampling tissues from different host species and sampling a wider geographical area, to bring light to this question.

## 4. Materials and Methods

### 4.1. P. salmonis Strains and Growth Conditions 

The strains used in this study are LF-89 (ATCC VR-1361), EM-90 (kindly donated by Dr. Sergio Marshall) and nine field isolates (isolated by Etecma, Puerto Montt, Chile), which are named here as PSCGR01, CGR02, Ps8079A, Ps2192A, Ps8942B, Ps11091B, Ps12201A, Ps18627A and Ps19647A. Further, strains NVI5692, NVI5892 and NVI5896 (kindly donated by Dr. Hanne Winther-Larsen) were also part of this work. LF-89, EM-90, PSCGR01, CGR02, Ps8079A, Ps8942B and Ps11091B were previously sequenced by our group, and their genome sequences are available at the NCBI Genome database under the accession numbers SAMN02469424, SAMN10437718, SAMN04309069, SAMN04309076, SAMN13898195, SAMN13898197 and SAMN13898198, respectively. The genogroup of the other four strains was characterized in this study. The strains were stored in cryopreservation vials at −80 °C and were revived by streaking a loopful of the frozen culture onto an Austral-SRS agar plate [23,24] and incubated at 18 °C aerobically for four days. Subcultures were maintained on the same medium and under the same conditions as described above. For the DNA extraction, *P. salmonis* cultures were inoculated by picking a fresh colony from an Austral-SRS agar plate into 5 mL of an Austral-SRS medium. *P. salmonis* cultures were grown at 18 °C with shaking at 180 rpm [25]. After 4 days, the cultures were diluted to an optical density (OD_600_) of 0.05 in 5 mL of an Austral-SRS medium and incubated at 18 °C. The absorbance was measured periodically in an Infinite^®^ 200 PRO NanoQuant (Tecan^®^) until the mid-exponential growth phase was reached. 

### 4.2. 16S rDNA Sequences and Restriction Enzymes Bioinformatics Selection

The 16S rDNA sequences of *P. salmonis* were retrieved from the NCBI_Genome database [50] and complemented with the 16S rDNA sequences of *P. salmonis* from Greengenes [51], Ribosomal Database Project [52] and SILVA [53]. To maximize the probability of finding differential restriction sites among the *P. salmonis* genogroups, we selected only those sequences that contain the 16S rRNA gene universal primers 27F and 1492R [54]. A phylogenetic analysis based on the 16S rRNA gene sequences showed that 38 out of 76 sequences (50%) were assigned to the EM-90-like genogroup and 38 out of 76 sequences (50%) to the LF-89-like genogroup (data not shown). Each sequence was trimmed into a fragment flanked by two conserved regions corresponding to the primers’ sites using reads_fasta and find_adaptor scripts of the bioinformatics suit Biopieces version 0.51. To obtain the restriction patterns, each trimmed sequence was in silico digested using the script Restrict from EMBOSS Suit version 6.3.1 with the following parameters: snucleotide1, sitelen = 4, rformat = table, and enzymes = enzymes.txt, according to Mandakovic et al. [18]. We selected 650 restriction enzymes that were commercially available in the REBASE database [55], and 152 out of 650 restriction enzymes digested the 76 *P. salmonis* 16S rDNA sequences that were previously selected. To select those restriction enzymes that generate easily differentiable restriction patterns between the LF-89-like and EM-90-like genogroups of *P. salmonis*, we designed a perl script that compares the bands of the digestion patterns produced by each of the 152 enzymes between the genogroups. If the digestion of the 16S rDNA sequences of a strain belonging to the EM-90-like genogroup produced a banding pattern that matched exactly that of an LF-89-like strain, and there were no additional bands that enabled to differentiate between the digestion patterns, then the enzyme was discarded (bad enzymes in Figure 1A). All enzymes that were not labeled as bad (good enzymes, N = 7) were manually revised by analyzing the digestion patterns generated by each of the seven selected enzymes. 

### 4.3. DNA Extraction from P. salmonis Strains

Bacterial DNA was purified from 1 mL of mid-exponential phase growth cultures using the DNeasy Blood and Tissue Kit for DNA (Qiagen). The bacterial culture samples were centrifuged for 10 min at 8000× *g*, supernatants were discarded and the pellets were disrupted according to the manufacturer’s instructions. The pellets were resuspended in 200 µL of PBS and 25 µL of proteinase K and 200 µL of AL buffer were added, and then they were incubated at 56 °C for 2 h. DNA was isolated using the DNeasy Mini spin columns. The purified DNA, dissolved in an AE buffer, was quantified with a NanoQuant Spectrophotometer (Tecan Technologies) and the DNA integrity was verified using agarose gel electrophoresis. The DNA was stored at −20 °C until used. 

### 4.4. Fish Samples Collection, DNA Extraction from Fish Tissues and P. salmonis Detection

The fish samples were collected according to the requirements of the Specific Sanitary Program for Surveillance and Control of Piscirickettsiosis implemented by the Chilean Fisheries and Aquaculture Center (Sernapesca, Valparaíso, Chile www.sernapesca.cl), which consists of periodic diagnostic tests to identify the presence of *P. salmonis* in fish from farm centers. The tissues were extracted from fresh mortalities of the day (less than 12 h). Using a scalpel, a cut was made in the lateral zone, and then five mm^3^ pieces of brain, liver and/or muscle were dissected from the fish. The organs were placed in a tube with 70% ethanol in a 1:10 ratio (sample/ethanol). The samples were kept at 4 ± 0.5 °C or lower temperatures. The total DNA was extracted from the fish tissue samples using the E.Z.N.A Tissue DNA Kit (Omega Bio-Tek, Norcross, GE, USA). Briefly, 30 mg of each tissue was homogenized and transferred to a microcentrifuge tube containing 200 μL of TL buffer, OB protease solution was added and the samples were incubated at 55 °C in a shaking water bath for 3 h. Then, the samples were centrifuged at 12,000× *g*, and the supernatant was transferred to a new tube. After the BL buffer was added, the mix was incubated at 70 °C for 10 min and finally 220 µL of 100% ethanol was added. The samples were transferred to the HiBind^®^ DNA Mini Column and centrifuged at maximum speed for 1 min at 12,000× *g*. After the sequential addition of the HBC and DNA wash buffer followed by centrifugation, the DNA was obtained by elution with 200 μL of elution buffer previously heated to 70 °C. The concentration and purity were measured using a NanoQuant Spectrophotometer (Tecan Technologies, Männedorf, Switzerland). 

The initial detection of *P. salmonis* in the field samples was carried out by amplification of the 23S rRNA gene from the genomic DNA using primers and probes described in Corbeil et al. [28]. The reaction was carried out in 20 µL containing 5 µL of Light cycler 480 probes Master, 1 µM of the Psal forward primer (5′-TCTGGGAAGTGGGATAGA-3′), 1 µM of the Psal reverse primer (5′-TCCCGACCTACTTGTTCATC-3′), 2 µM of probe (5′ 6FAM-TGATAGCCCCGTACACGAAAC GCATA-MGB NFQ 3′), 1.5 µL of nuclease-free water and 2.5 µL of DNA. The following PCR conditions were used: 95 °C for 10 min, followed by 45 cycles of 95 °C for 15 s, 60 °C for 1 min and 72 °C for 10 s. The samples were considered positive for *P. salmonis* if amplification took place before cycle 36.7.

### 4.5. 16S rRNA Gene Amplification and Sequencing

The 16S rRNA gene was amplified from 50 ng of genomic DNA using the universal primers 27F (5′-AGAGTTTGATCCTGGCTCAG-3′) and 1492R (5′-CGGTTACCTTGTTACGACTT-3′) [54]. The reactions were carried out in 25 μL volumes, which contained 12.5 μL of GoTaq^®^ Green Master Mix (Promega, Madison, WI, USA), 10 µM of each primer and 8.5 μL of nuclease-free water. The following PCR conditions were used: initial denaturation at 95 °C for 10 min, 30 cycles of 95 °C for 60 s, 60 °C for 30 s and 72 °C for 90 s, with a final extension at 72 °C for 10 min. The amplification products were visualized on 1% agarose gels by ethidium bromide staining and UV illumination. The PCR products from the 16S rRNA gene amplification of the *P. salmonis* isolates were purified and sequenced in Macrogen (Seoul, Korea). The sequences of the 16S rRNA genes were deposited in GenBank and the accession numbers are given in Supplementary Tables S2 and S3. 

### 4.6. Phylogenetic Analyses of 16S rRNA Gene

The quality of the 16S rRNA gene sequences was corroborated using the BioEdit v7.0.5.3 software [56]. The sequences were manually filtered and trimmed to a length of 1354 bp. Afterwards, sequences were aligned using the MAFFT v7.313 software [57] with the default (FFT-NS-2) algorithm, subsequently trimmed to a length of 1354 bp and realigned using the G-INS-I algorithm. The phylogenetic analyses were conducted using the MEGA X software [58], with the following settings: Maximum likelihood tree, Tamura–Nei model, with 1000 bootstrap replications. The trees were visualized and annotated using the FigTree v1.4.3 software (http://tree.bio.ed.ac.uk/software/figtree).

### 4.7. PCR-RFLP Assays of P. salmonis Strains and Field Samples

The 16S rRNA gene was amplified from the genomic DNA samples of 13 *P. salmonis* strains and 57 fish tissues (field samples) as described in Section 4.5. Initially, the presence and purity of *P. salmonis* in the field samples were verified as described in Mandakovic et al. [18]. Briefly, 5 µL of the amplification products was digested using the restriction enzyme *PmaC*I (FastDigest Eco72I, ThemoFisher) for 15 min at 37 ℃ followed by agarose gel electrophoresis of the restriction fragments on a 2% gel stained with ethidium bromide. The fragments were visualized in a UV transilluminator and photographed. Then, the PCR amplification products from the *P. salmonis* strains and field samples were subjected to a *Xap*I enzymatic digestion. The reactions were carried out in 15 μL volumes, which contained 5 μL of the PCR product, 1.5 μL of enzyme buffer, 1 U of each enzyme and 8 μL of nuclease-free water, for 30 min at 37 °C. The restriction fragments were visualized by electrophoresis in 2% agarose gels stained with 0.5 µg/mL of ethidium bromide for 30 min at room temperature. 

### 4.8. Sequencing of Bacterial 16S rDNA Amplicons

The total DNA (fish + pathogen DNA) extracted from the field samples was amplified using a bacteria-specific primer set with 27F (5′-GAGTTTGATCMTGGCTCAG-3′) and 519R (5′-GWATTACCGCGGCKGCTG-3′), flanking the hypervariable regions V1–V3 of the 16S rRNA gene [59] with a barcode on the forward primer. Amplification was carried out using the Qiagen Kit HotStarTaq Plus Master Mix under the conditions described in Mandakovic et al. (2018) [60]. After amplification, the PCR products were visualized in a 2% agarose gel to determine the success of the amplification and the relative intensity of the bands. At this point, the three barcoded samples for the microbial analyses were pooled together in equal proportions based on their molecular weight and DNA concentrations. Pooled samples were purified using calibrated Agencourt AMPure XP beads and used to prepare the DNA libraries following the Illumina TruSeq DNA library preparation protocol. The sequencing was performed at the molecular research DNA laboratory (Shallowater, TX, USA) on an Illumina MiSeq platform in an overlapping 2 × 300 bp configuration with a minimum throughput of 20,000 reads per sample. 

### 4.9. Analysis of 16S rDNA Amplicon Sequencing

The 16S rDNA raw amplicon sequences were processed and analyzed following the previously described protocols [61,62]. Briefly, sequences were joined (overlapping pairs) and grouped by samples following the barcodes, and then the barcodes were removed and the sequences <150 bp or with ambiguous base calls were discarded. The sequences were filtered using the USEARCH clustering algorithm at a 4% sequence divergence to remove chimeras and clusters consisting of only one sequence (i.e., singletons) [63,64]. Finally, the sequences were quality filtered with Mothur v.1.22.2 [65] with the minimal quality average set to 30. The sequences were analyzed with the software quantitative insights into microbial ecology (QIIME) v1.9.1, [66]. Briefly, we used the QIIME script “pick_closed_reference_otus.py” to extract all the 16S rRNA reads from the amplicon data that matched the Silva r16S database release 132 [67] at 97% similarity or 3% divergence, with the taxonomy of the resulting operational taxonomic units (OTUs) assigned directly from the closest reference sequence match. The OTU picking process was done with USEARCH v6.1.544 [63,64] using the QIIME default parameter values (-s0.97 –z True –max_accepts 1 –max_rejects 8 –word_length 8 –minlen 64 – usearch61_sort_method abundance). OTUs unassigned or assigned to mitochondria and chloroplast were removed. To characterize the microbial diversity patterns, we calculated the alpha OTU diversity by randomly sub-sampling (without replacement) each sample using the alpha_rarefaction.py script in QIIME. Rarefaction curves for each of these metrics were obtained by serial subsampling in increments of 1300 sequences and 10 iterations per increment to the standardized 13,000 sequences per sample. This number represented the lowest number of curated sequences obtained across our samples. The OTUs’ identification numbers, abundance and taxonomy are specified in Table S4. All 16S rDNA sequence data used in this study are deposited in the Sequence Read Archive (SRA) of the National Center for Biotechnology Information (NCBI) under the BioProject accession number PRJNA613955.

## Supplementary Materials

The following are available online at https://www.mdpi.com/2076-0817/9/5/358/s1, Table S1: Features of the seven selected restriction enzymes, Figure S1: Multiple alignment of the 16S rDNA sequences of the *P. salmonis* strains, Table S2: *P. salmonis* strains used in this study, Figure S2: PCR amplification of the 16S rDNA sequences, Figure S3: PCR-RFLP analysis of the field samples with mixtures of bacterial DNA, Table S3: field samples analyzed in this study, Table S4: taxonomy and average relative abundance of each OTU in 10 fish samples.

## References

[1] Fryer J.L., Hedrick R.P. (2003). Review piscirickettsia salmonis: A Gram-negative intracellular bacterial pathogen of fish. J. Fish. Dis..

[2] Groff J., LaPatra S. (2000). Infectious diseases impacting the commercial culture of salmonids. J. Appl. Aquacult..

[3] Sernapesca (2019). Informe Sanitario De Salmonicultura En Centros Marinos Año 2018.

[4] Fryer J.L., Lannan C.N., Giovannoni S.J., Wood N.D. (1992). *Piscirickettsia salmonis* gen. nov., sp. nov. the causative agent of an epizootic disease in salmonid fishes. Int. J. Syst. Bacteriol..

[5] Fryer J.L., Lannan C.N., Giovannoni S.J., Wood N.D. (1990). *Piscirickettsia salmonis* gen. nov., sp. nov., the causative agent of an epizootic disease in salmonid fishes. Fish Pathol..

[6] Cvitanich J.D., Garate O.N., Smith C.E. (1991). The isolation of a rickettsia-like organism causing disease and mortality in Chilean salmonids and its confirmation by Koch’ s postulate. J. Fish Dis..

[7] Branson E.J., Nieto Diaz-Munoz D. (1991). Description of a new disease condition occurring in farmed coho salmon, *Oncorhynchus kisuteh* (Walbaum), in South America. J. Fish. Dis..

[8] Fryer J.L., Lannan C.N., Garcés L.H., Larenas J.J., Smith P.A. (1990). Isolation of a rickettsiales-like organism from diseased coho salmon (*Oncorhynchus kisutch*) in Chile. Fish. Pathol..

[9] Brocklebank J.R., Speare D.J., Armstrong R.D., Evelyn T. (1992). British Columbia. Septicemia suspected to be caused by a ricksettia-like agent in farmed Atlantic salmon. Can. Vet. J..

[10] Corbeil S., Hyatt A.D., Crane M.S.J. (2005). Characterisation of an emerging rickettsia-like organism in Tasmanian farmed Atlantic salmon *Salmo salar*. Dis. Aquat. Organ..

[11] Rozas M., Enríquez R. (2014). *Piscirickettsiosis* and *Piscirickettsia salmonis* in fish: A review. J. Fish. Dis..

[12] Birrell J., Mitchell S., Bruno D.W. (2003). Piscirickettsia salmonis in farmed Atlantic salmon, *Salmo solar* in Scotland. Bull. Eur. Assoc. Fish. Pathol..

[13] Olsen A.B., Melby H.P., Speilberg L., Evensen Ø., Håstein T. (1997). *Piscirickettsia salmonis* infection in Atlantic salmon *Salmo salar* in Norway-epidemiological, pathological and microbiological findings. Dis. Aquat. Organ..

[14] Jones S.R.M., Markham F.R.J., Groman D.B., Cusack R.R. (1998). Virulence and antigenic characteristics of a cultured Rickettsiales-like organism isolated from farmed Atlantic salmon *Salmo salar* in eastern Canada. Dis. Aquat. Organ..

[15] House M., Bartholomew J., Winton J., Fryer J. (1992). Relative virulence of three isolates of *Piscirickettsia salmonis* for coho salmon *Oncorhynchus Kisutch*. Dis. Aquat. Organ..

[16] Mauel M.J., Giovannoni S.J., Fryer J.L. (1999). Phylogenetic analysis of *Piscirickettsia salmonis* by 16S, internal transcribed spacer (ITS) and 23s ribosomal DNA sequencing. Dis. Aquat. Organ..

[17] Bohle H., Henríquez P., Grothusen H., Navas E., Sandoval A., Bustamante F., Bustos P., Mancilla M. (2014). Comparative genome analysis of two isolates of the fish pathogen *Piscirickettsia salmonis* from different hosts reveals major differences in virulence-associated secretion systems. Genome Announc..

[18] Mandakovic D., Glasner B., Maldonado J., Aravena P., González M., Cambiazo V., Pulgar R. (2016). Genomic-based restriction enzyme selection for specific detection of *Piscirickettsia salmonis* by 16S rDNA PCR-RFLP. Front. Microbiol..

[19] Otterlei A., Øyvind J., Brevik D.J., Duesund H., Sommerset I., Frost P., Mendoza J., McKenzie P., Nylund A., Apablaza P. (2016). Phenotypic and genetic characterization of *Piscirickettsia salmonis* from Chilean and Canadian salmonids. BMC Vet. Res..

[20] Nourdin-Galindo G., Sánchez P., Molina F.C., Espinoza-Rojas D.A., Oliver C., Ruiz P., Vargas-Chacoff L., Cárcamo J.G., Figueroa J.E., Mancilla M. (2017). Comparative pan-genome analysis of *Piscirickettsia salmonis* reveals genomic divergences within genogroups. Front. Cell. Infect. Microbiol..

[21] Saavedra J., Hernandez N., Osses A., Castillo A., Cancino A., Grothusen H., Navas E., Henriquez P., Bohle H., Bustamante F. (2017). Prevalence, geographic distribution and phenotypic differences of *Piscirickettsia salmonis* EM-90-like isolates. J. Fish. Dis..

[22] Rozas-Serri M., Ildefonso R., Peña A., Enríquez R., Barrientos S., Maldonado L. (2017). Comparative pathogenesis of piscirickettsiosis in Atlantic salmon (*Salmo salar* L.) post-smolt experimentally challenged with LF-89-like and EM-90-like *Piscirickettsia salmonis* isolates. J. Fish. Dis..

[23] Mauel M.J., Ware C., Smith P.A. (2008). Culture of *Piscirickettsia salmonis* on enriched blood agar. J. Vet. Diagn. Investig..

[24] Mikalsen J., Skjaervik O., Wiik-Nielsen J., Wasmuth M.A., Colquhoun D.J. (2008). Agar culture of *Piscirickettsia salmonis*, a serious pathogen of farmed salmonid and marine fish. FEMS Microbiol. Lett..

[25] Vera T., Isla A., Cuevas A., Figueroa J. (2012). A new liquid medium for the pathogen *Piscirickettsia salmonis* T. Arch. Med. Vet..

[26] Mauel M.J., Giovannoni S.J., Fryer J.L. (1996). Development of polymerase chain reaction assays for detection, identification, and differentiation of *Piscirickettsia salmonis*. Dis. Aquat. Org..

[27] Marshall S., Heath S., Henríquez V., Orrego C. (1998). Minimally invasive detection of *Piscirickettsia salmonis* in cultivated salmonids via the PCR. Appl. Environ. Microbiol..

[28] Corbeil S., McColl K.A., Crane M.S.J. (2003). Development of a TaqMan quantitative PCR assay for the identification of *Piscirickettsia salmonis*. Bull. Eur. Assoc. Fish. Pathol..

[29] Karatas S., Mikalsen J., Steinum T.M., Taksdal T., Bordevik M., Colquhoun D.J. (2008). Real time PCR detection of *Piscirickettsia salmonis* from formalin-fixed paraffin-embedded tissues. J. Fish Dis..

[30] Lannan C., Ewing S., Fryer J. (1991). A fluorescent antibody test for detection of the rickettsia causing disease in chilean salmonids. J. Aquat. Anim. Health.

[31] Reiling N., Homolka S., Walter K., Brandenburg J., Niwinski L., Ernst M., Herzmann C., Lange C., Diel R., Ehlers S. (2013). Clade-specific virulence patterns of mycobacterium tuberculosis complex strains in human primary macrophages and aerogenically infected mice. MBio.

[32] Sousa P.S., Silva I.N., Moreira L.M., Veríssimo A., Costa J. (2018). Differences in virulence between *Legionella pneumophila* isolates from human and non-human sources determined in Galleria mellonella infection model. Front. Cell. Infect. Microbiol..

[33] Tamura K., Nei M. (1993). Estimation of the number of nucleotide substitutions in the control region of mitochondrial DNA in humans and chimpanzees. Mol. Biol. Evol..

[34] Isla A., Saldarriaga-Córdoba M., Fuentes D.E., Albornoz R., Haussmann D., Mancilla-Schulz J., Martínez A., Figueroa J., Avendaño-Herrera R., Yáñe A. (2019). Multilocus sequence typing detects new Piscirickettsia salmonis hybrid genogroup in Chilean fish farms: Evidence for genetic diversity and population structure. J. Fish. Dis..

[35] Hallin M., Deplano A., Struelens M.J. (2012). Molecular typing of bacterial pathogens: A tool for the epidemiological study and control of infectious diseases. New Frontiers of Molecular Epidemiology of Infectious Diseases.

[36] Karlsen C., Vanberg C., Mikkelsen H., Sørum H. (2014). Co-infection of *Atlantic salmon* (*Salmo salar*), by *Moritella viscosa* and *Aliivibrio wodanis*, development of disease and host colonization. Vet. Microbiol..

[37] Loch T.P., Scribner K., Tempelman R., Whelan G., Faisal M. (2012). Bacterial infections of *Chinook salmon*, *Oncorhynchus tshawytscha* (Walbaum), returning to gamete collecting weirs in Michigan. J. Fish. Dis..

[38] Zarkasi K.Z., Abell G.C.J., Taylor R.S., Neuman C., Hatje E., Tamplin M.L., Katouli M., Bowman J.P. (2014). Pyrosequencing-based characterization of gastrointestinal bacteria of Atlantic salmon (*Salmo salar* L.) within a commercial mariculture system. J. Appl. Microbiol..

[39] De Bruijn I., Liu Y., Wiegertjes G.F., Raaijmakers J.M. (2018). Exploring fish microbial communities to mitigate emerging diseases in aquaculture. FEMS Microbiol. Ecol..

[40] Larsen A., Tao Z., Bullard S.A., Arias C.R. (2013). Diversity of the skin microbiota of fishes: Evidence for host species specificity. FEMS Microbiol. Ecol..

[41] Galbraith H., Iwanowicz D., Spooner D., Iwanowicz L., Keller D., Zelanko P., Adams C. (2018). Exposure to synthetic hydraulic fracturing waste influences the mucosal bacterial community structure of the brook trout (*Salvelinus fontinalis*) epidermis. AIMS Microbiol..

[42] Navarrete P., Espejo R.T., Romero J. (2009). Molecular analysis of microbiota along the digestive tract of juvenile atlantic salmon (*Salmo salar* L.). Microb. Ecol..

[43] Zhou Z., Liu Y., Shi P., He S., Yao B., Ringø E. (2009). Molecular characterization of the autochthonous microbiota in the gastrointestinal tract of adult yellow grouper (*Epinephelus awoara*) cultured in cages. Aquaculture.

[44] Egerton S., Culloty S., Whooley J., Stanton C., Ross R.P. (2018). The gut microbiota of marine fish. Front. Microbiol..

[45] Salgado-Miranda C., Palomares E., Jurado M., Marín A., Vega F., Soriano-Vargas E. (2010). Isolation and distribution of bacterial flora in farmed rainbow trout from Mexico. J. Aquat. Anim. Health.

[46] Sevellec M., Pavey S.A., Boutin S., Filteau M., Derome N., Bernatchez L. (2014). Microbiome investigation in the ecological speciation context of lake whitefish (*Coregonus clupeaformis*) using next-generation sequencing. J. Evol. Biol..

[47] Gomez-Gil B., Fajer-Avila E., García-Vargas F. (2007). Vibrios of the spotted rose snapper *Lutjanus guttatus* Steindachner, 1869 from northwestern Mexico. J. Appl. Microbiol..

[48] Meron D., Davidovich N., Ofek-Lalzar M., Berzak R., Scheinin A., Regev Y., Diga R., Tchernov D., Morick D. (2020). Specific pathogens and microbial abundance within liver and kidney tissues of wild marine fish from the eastern Mediterranean sea. Microb. Biotechnol..

[49] Tort L. (2011). Stress and immune modulation in fish. Dev. Comp. Immunol..

[50] Clark K., Karsch-Mizrachi I., Lipman D.J., Ostell J., Sayers E.W. (2016). GenBank. Nucleic Acids Res..

[51] DeSantis T.Z., Hugenholtz P., Larsen N., Rojas M., Brodie E.L., Keller K., Huber T., Dalevi D., Hu P., Andersen G.L. (2006). Greengenes, a chimera-checked 16S rRNA gene database and workbench compatible with ARB. Appl. Environ. Microbiol..

[52] Cole J.R., Wang Q.E., Cardenas J., Fish B., Chai R.J., Farris A.S., Kulam-Syed-Mohideen D.M., McGarrell T., Marsh G., Garrity M. (2009). The Ribosomal database project: Improved alignments and new tools for rRNA analysis. Nucleic Acids Res..

[53] Pruesse E., Quast C., Knittel K., Fuchs B.M., Ludwig W., Peplies J., Glöckner F.O. (2007). SILVA: A comprehensive online resource for quality checked and aligned ribosomal RNA sequence data compatible with ARB. Nucleic Acids Res..

[54] Jiang H., Dong H., Zhang G., Yu B., Chapman L.R., Fields M.W. (2006). Microbial diversity in water and sediment of lake Chaka, an athalassohaline lake in northwestern China. Appl. Environ. Microbiol..

[55] Roberts R.J., Vincze T., Posfai J., Macelis D. (2015). REBASE—A database for DNA restriction and modification: Enzymes, genes and genomes. Nucleic Acids Res..

[56] Hall T. (1999). BioEdit: A user-friendly biological sequence alignment editor and analysis program for windows 95/98/NT. Nucleic Acids Sumpos..

[57] Katoh K., Standley D.M. (2013). MAFFT multiple sequence alignment software version 7: Improvements in performance and usability. Mol. Biol. Evol..

[58] Kumar S., Stecher G., Li M., Knyaz C., Tamura K. (2018). MEGA X: Molecular evolutionary genetics analysis across computing platforms. Mol. Biol. Evol..

[59] Turner S., Pryer K.M., Miao V.P., Palmer J.D. (1999). Investigating deep phylogenetic relationships among cyanobacteria and plastids by small subunit rRNA sequence analysis. J. Eukaryot. Microbiol..

[60] Mandakovic D., Maldonado J., Pulgar R., Cabrera P., Gaete A., Urtuvia V., Seeger M., Cambiazo V., González M. (2018). Microbiome analysis and bacterial isolation from Lejía lake soil in Atacama desert. Extremophiles.

[61] Dowd S.E., Callaway T.R., Wolcott R.D., Sun Y., McKeehan T., Hagevoort R.G., Edrington T.S. (2008). Evaluation of the bacterial diversity in the feces of cattle using 16S rDNA bacterial tag-encoded FLX amplicon pyrosequencing (bTEFAP). BMC Microbiol..

[62] Handl S., Dowd S.E., Garcia-Mazcorro J.F., Steiner J.M., Suchodolski J.S. (2011). Massive parallel 16S rRNA gene pyrosequencing reveals highly diverse fecal bacterial and fungal communities in healthy dogs and cats. FEMS Microbiol. Ecol..

[63] Edgar R.C. (2010). Search and clustering orders of magnitude faster than BLAST. Bioinformatics.

[64] Edgar R.C., Haas B.J., Clemente J.C., Quince C., Knight R. (2011). UCHIME improves sensitivity and speed of chimera detection. Bioinformatics.

[65] Schloss P.D., Westcott S.L., Ryabin T., Hall J.R., Hartmann M., Hollister E.B., Lesniewski R.A., Oakley B.B., Parks D.H., Robinson C.J. (2009). Introducing mothur: Open-source, platform-independent, community-supported software for describing and comparing microbial communities. Appl. Environ. Microbiol..

[66] Caporaso J.G., Kuczynski J., Stombaugh J., Bittinger K., Bushman F.D., Costello E.K., Fierer N., Peña A.G., Goodrich J.K., Gordon J.I. (2010). QIIME allows analysis of high-throughput community sequencing data. Nat. Methods.

[67] Quast C., Pruesse E., Yilmaz P., Gerken J., Schweer T., Yarza P., Peplies J., Glöckner F.O. (2012). The SILVA ribosomal RNA gene database project: Improved data processing and web-based tools. Nucleic Acids Res..

