# Supplementary material for: PCR-RFLP Detection and Genogroup Identification of Piscirickettsia salmonis in Field Samples
Source: Pathogens. 2020 May 8;9(5):358. doi: 10.3390/pathogens9050358 (PMC7281544; doi:10.3390/pathogens9050358)
Supplement: Supplementary file 1 [file pathogens-09-00358-s001.zip › Supplementary material/Figure S1.docx]

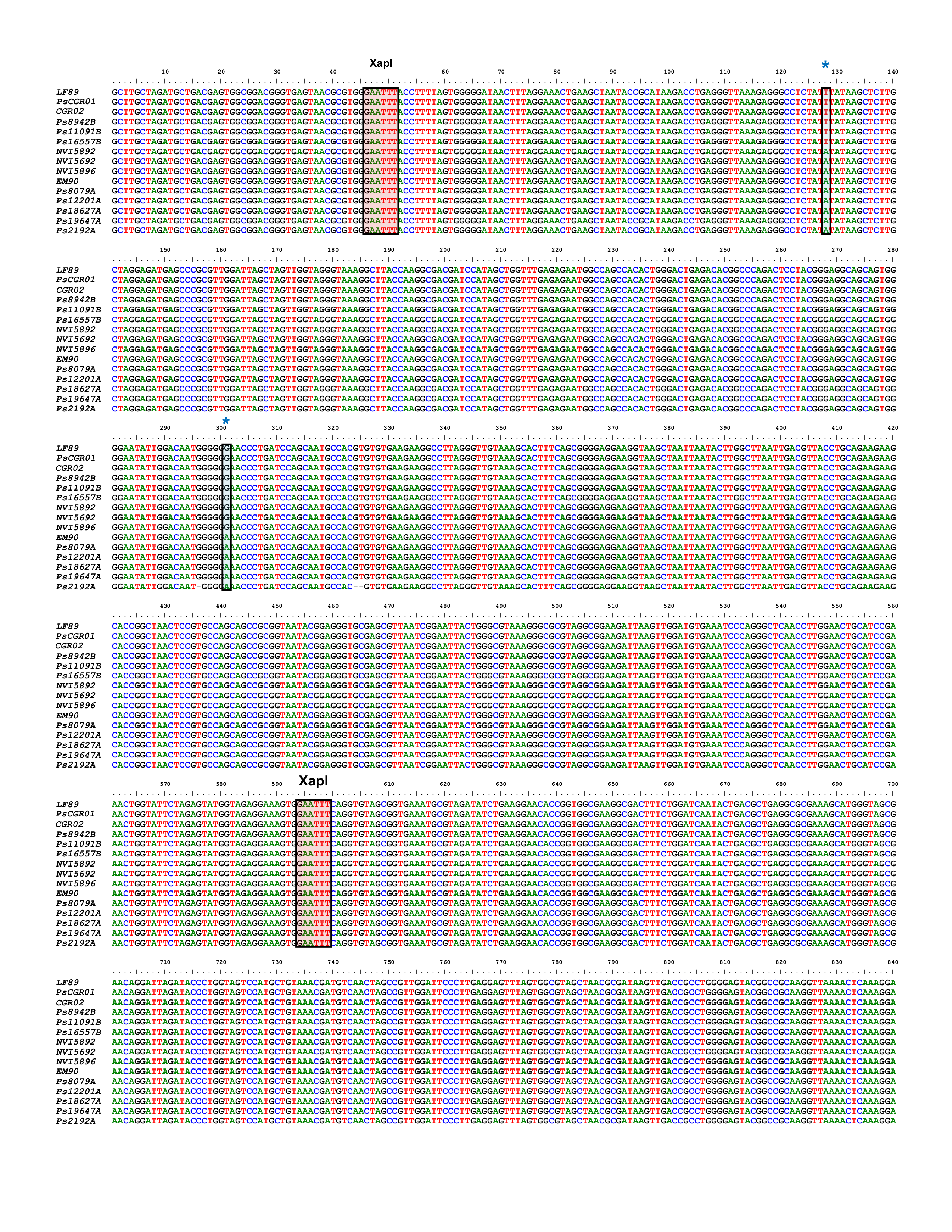


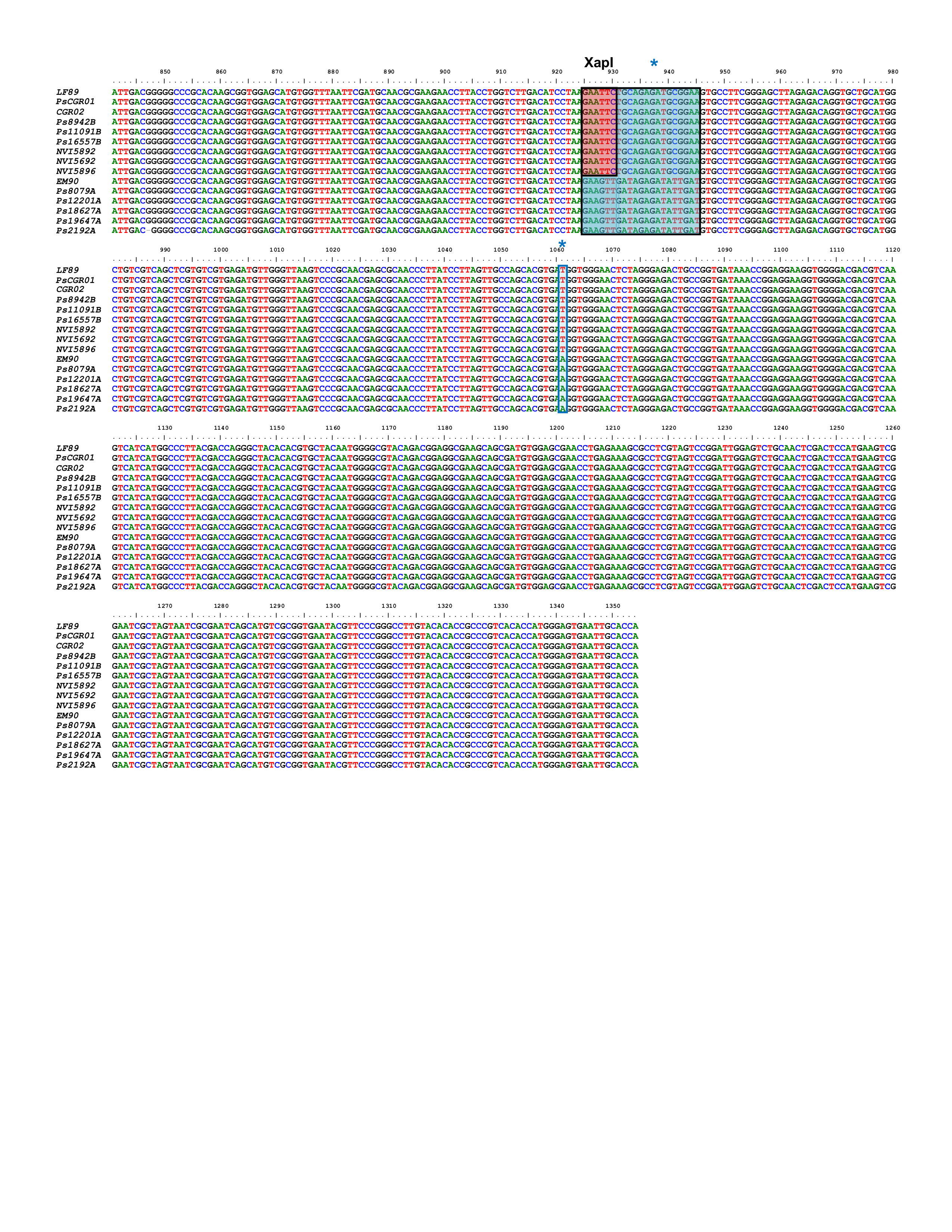


**Figure S1:** Multiple alignment of the 16S rDNA sequences of *P. salmonis* strains. *Xap*I restriction sites are boxed and shaded in red. Nucleotides that differ between LF-89-like and EM-90-like genogroups are shaded in green and indicated with an asterisk.
