# Supplementary material for: PCR-RFLP Detection and Genogroup Identification of Piscirickettsia salmonis in Field Samples
Source: Pathogens. 2020 May 8;9(5):358. doi: 10.3390/pathogens9050358 (PMC7281544; doi:10.3390/pathogens9050358)
Supplement: Supplementary file 1 [file pathogens-09-00358-s001.zip › Supplementary material/Figure S2.docx]

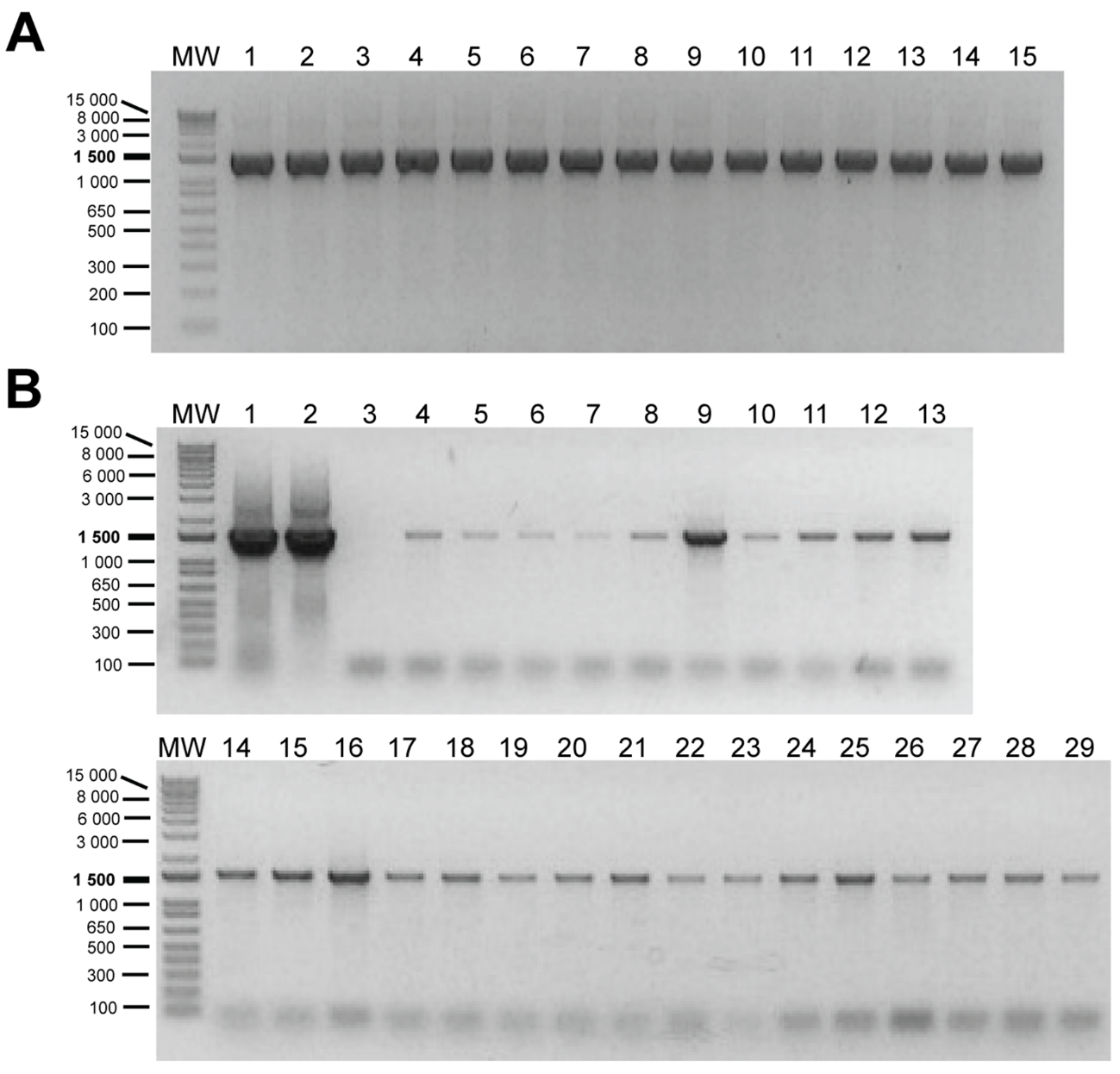


**Figure S2.** PCR amplification of 16S rDNA sequences. A, 16S rDNA fragment amplified from *P. salmonis* strains. Lanes 1: LF89, 2: PSCGR01, 3: CGR02, 4: Ps8942, 5: Ps11091, 6: Ps16557, 7: EM-90, 8: Ps8079, 9: Ps2192, 10: Ps12201, 11: Ps18627, 12: Ps19647, 13: NVI5692, 14: NVI5892, 15: NVI5896. B, 16S rDNA fragment amplified from field samples. Lanes 1: LF89, 2: EM-90, 3: no template control, 4: Ps18250-3, 5: Ps18700-3, 6: Ps18700-1, 7: Ps18662-3, 8: Ps18662-2, 9: Ps18623-3, 10: Ps18623-2, 11: Ps18621-7, 12: Ps18560-1, 13: Ps18437-1, 14: Ps18258-3, 15: Ps16557-1, 16: Ps12093-5, 17: Ps19654, 18: Ps18301-9, 19: Ps18316-3, 20: Ps18729, 21: Ps19654-2, 22: Ps14650, 23: Ps18766-1, 24: Ps18426-2, 25: Ps18426-3, 26: Ps18429-3, 27: Ps18432-3, 28: Ps18432-5, 29: Ps18448-2. MW: 1 k plus p DNA Ladder (Invitrogen).
