# Supplementary material for: PCR-RFLP Detection and Genogroup Identification of Piscirickettsia salmonis in Field Samples
Source: Pathogens. 2020 May 8;9(5):358. doi: 10.3390/pathogens9050358 (PMC7281544; doi:10.3390/pathogens9050358)
Supplement: Supplementary file 1 [file pathogens-09-00358-s001.zip › Supplementary material/Figure S3.docx]

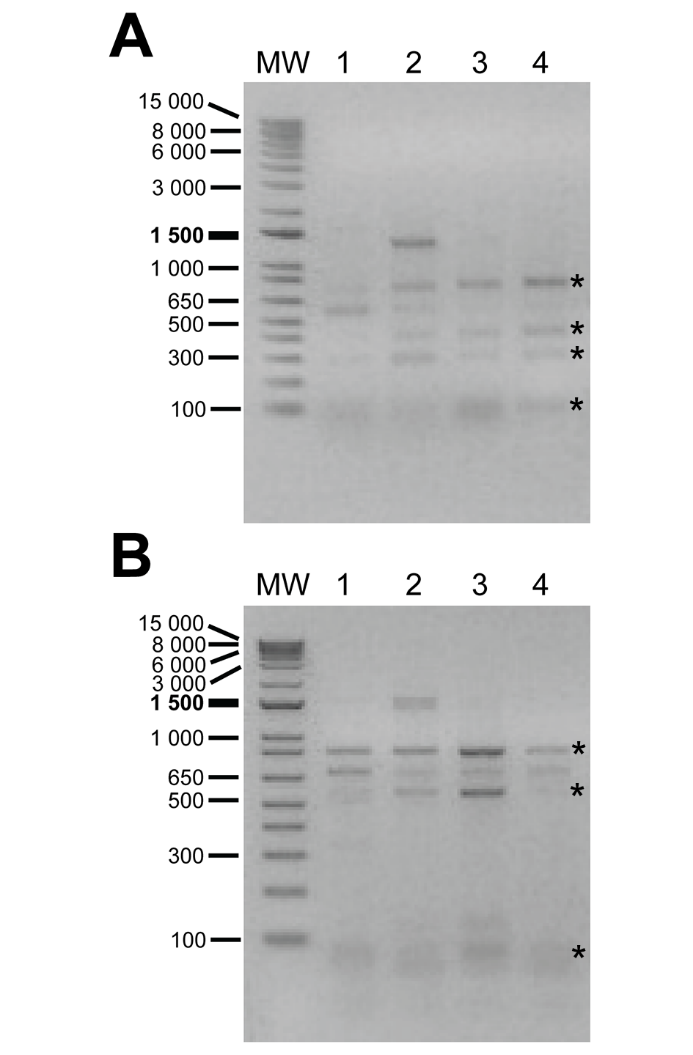


**Figure S3:** PCR-RFLP analysis of field samples with mixtures of bacterial DNA. Representative images of 16S rDNA gene digestion patterns with enzyme *PmaC*I (A) and *XapI* (B) of four field samples. The expected bands after *PmaC*I or *XapI* digestion are indicated by asterisks. *PmaCI* enzyme generates four bands of 732, 395, 280 and 97 bp, in the 16S rDNA gene of *P. salmonis*. *XapI* enzyme generates three bands in the 16S rDNA gene of EM-90-like strains (840, 548 and 119 bp). In this case, only *P. salmonis* belonging to the EM-90-like genogroup was detected in the four selected field samples.
