# Supplementary material for: PCR-RFLP Detection and Genogroup Identification of Piscirickettsia salmonis in Field Samples
Source: Pathogens. 2020 May 8;9(5):358. doi: 10.3390/pathogens9050358 (PMC7281544; doi:10.3390/pathogens9050358)
Supplement: Supplementary file 1 [file pathogens-09-00358-s001.zip › Supplementary material/Table S1.docx]

| **Table S1:** Features of the seven selected restriction enzymes. | | |  |
| --- | --- | --- | --- |
| **Enzyme** (isoschizomer) | **Length (bp) and number (n) of bands**^1^ | | |
|  | **EM-90 like** | **LF-89 like** | |
| *XapI (ApoI)* | 840, 548, 119 (n=3) | 548, 509, 331, 119 (n=4) | |
| *SfaNI (BmsI, LweI)* | 563, 559, 313, 72 (n=4) | 563, 507, 313, 72, 52 (n=5) | |
| *Hin4II (HpyAV)* | 400, 318, 260, 254, 94, 85, 42, 37, 17 (n=9) | 400, 318, 260, 254, 136, 85, 37, 17 (n=8) | |
| *MluCI (Sse9I, TasI)* | 465, 334, 245, 119, 116, 90, 82, 40, 16 (n=9) | 419, 334, 245, 119, 116, 90, 82, 46, 40, 16 (n=10) | |
| *AciI (BspACI, SsiI)* | 272, 208, 193, 158, 112, 103, 96, 83, 70, 61, 58, 35, 31, 17, 8, 2 (n=16) | 272, 208, 193, 112, 103, 96, 83, 79, 79, 70, 61, 58, 35, 31, 17, 8, 2 (n=17) | |
| *FaiI* | 372, 150, 144, 125, 122, 113, 106, 76, 67, 50, 36, 36, 34, 32, 29, 11, 2, 2 (n=18) | 372, 150, 144, 125, 122, 113, 106, 76, 67, 50, 36, 36, 34, 33, 32, 11 (n=16) | |
| *SetI* | 181, 135, 126, 114, 97, 77, 73, 66, 59, 52, 52, 51, 50, 42, 42, 41, 38, 33, 33, 31, 25, 21, 17, 11, 11, 8, 6, 5, 5, 5 (n=30) | 181, 135, 126, 114, 97, 77, 73, 66, 59, 52, 52, 51, 50, 42, 42, 41, 38, 33, 33, 31, 25, 21, 17, 11, 11, 8, 5, 5, 5 (n=29) | |

^1^ Predicted number and length of the fragments as result of *in silico* restriction digestions of EM-90 and LF-89-like genomes
